# Breast Cancer Mortality Trends and Predictions to 2030 and Its Attributable Risk Factors in East and South Asian Countries

**DOI:** 10.3389/fnut.2022.847920

**Published:** 2022-03-14

**Authors:** Sumaira Mubarik, Rajesh Sharma, Syeda Rija Hussain, Mujahid Iqbal, Nawsherwan, Xiaoxue Liu, Chuanhua Yu

**Affiliations:** ^1^Department of Epidemiology and Biostatistics, School of Public Health, Wuhan University, Wuhan, China; ^2^University School of Management and Entrepreneurship Delhi Technological University Delhi, New Delhi, India; ^3^Department of Health Sciences, Rawalpindi Medical University, Rawalpindi, Pakistan; ^4^Department of Psychology, School of Philosophy, Wuhan University, Wuhan, China; ^5^Institute of Cardiovascular Diseases, Xiamen University, Xiamen, China

**Keywords:** breast cancer mortality, prediction, Lee–Carter model, life expectancy, risk factors

## Abstract

**Background:**

Amidst the rising breast cancer burden in Asia, we aim to predict the future mortality risk due to breast cancer and identify the risk-attributable deaths for breast cancer among East and South Asian countries.

**Methods:**

We used country-level data to predict the trends in the next decade relating to female breast cancer mortality by employing data from 1990 to 2019 from the Global Burden of Disease 2019 study. We used the stochastic mortality modeling and prediction techniques to forecast the age-specific and risk-attributable breast cancer mortality trends at the regional and national levels of East and South Asia.

**Results:**

The number of deaths caused by the breast cancer is predicted to increase in East and South Asian countries in the next decade (2020–2030). Age-standardized death rate (ASDR) of breast cancer is predicted to increase by 7.0% from 9.20/100,000 (95% CI: 6.04–12.12) in 1990 to 9.88/100,000 (95% CI: 7.12–11.4) in 2030 in East Asia, and about 35% increase from 13.4/100,000 (95% CI: 9.21–16.02) in 1990 to 18.1/100,000 (95% CI: 13.23–21.10) in 2030 in South Asia. At the national level, the highest percent change in ASDR between 1990 and 2030 was reported in Pakistan (a 62% increase) and Nepal (a 47% increase). The highest percent change in breast cancer mortality between 2020 and 2030 for females of age group 80–84 years was observed in Pakistan [21.6, (95% CI, 20.6–94.7)], followed by Afghanistan [13.3 (4.0–80.8)], and Nepal [36.6 (11.1–125.7)] as compared to the other countries. In the females of aged 50–80 years, the predicted death rates were associated with high body mass index, high-fasting plasma glucose, and diet high in red meat, across the majority of countries under study. Furthermore, reductions in percent change in mortality rates occurred in several countries with increases in sociodemographic index (SDI), notably across high SDI countries.

**Conclusion:**

Breast cancer mortality risk varies substantially across East and South Asian countries with higher mortality risk in low/middle SDI countries. Early detection using screening, awareness among females and health workers, and cost-effective and timely treatment of patients with breast cancer is vital in stemming the tide of breast cancer in the next decade.

## Introduction

Developing countries and countries undergoing a westernized lifestyle in Asia may be facing a breast cancer epidemic in the near future due to rising breast cancer cases and deaths (1, 2). Breast cancer has been considered a disease of the Western World in the past but the situation has changed considerably in the past two decades and the disease is not a concern of developed countries alone, but is equally or even more damaging to several Asian countries. In Asia, breast cancer is the most common cancer and the second leading cause of cancer-related deaths among women, with Asia accounting for 39% of all breast cancer incident cases diagnosed globally (2). This rising breast cancer mortality across Asia is posing stress on the oncologic system and hampering an individual's quality of life (2). Previous literature has found better breast cancer survival rates in developed countries than in developing or least-developed ones (3). In 2012, deaths due to breast cancer amounted to 40.8% of the all-cancer deaths in females, ranking second behind lung cancer, illustrating breast cancer as a major health issue with alarming situations in Asia. The age-standardized mortality rate of breast cancer in Asia is 10.2/100,000, which is lower than the global level (12.9/100,000) and matching countries with a medium human development index (2). There were also large differences in breast cancer mortality rates across Asia, ranging from 8.6/100,000 in East Asia to 15.0/100,000 in South Asia in 2017 (4). It has been observed that the mortality-to-incidence ratio (MIR) of breast cancer is high in South-Central Asia and low in Eastern Asia (5).

Globally, an increase in breast cancer has been reported partly due to aging and population expansion (6, 7). Several modifiable risk factors such as lifestyle factors, obesity, excess alcohol consumption, smoking, no or reduced physical activity, and also unhealthy or insufficient diet, if addressed, might help in cancer control and prevention (8, 9). Westernized lifestyle and infection-related etiological factors also have contributions toward increased breast cancer incidence and mortality in developing and underdeveloped countries (2, 10).

Overall, Asia has forty-eight countries with almost 32% of the global female population and most of them are middle- and low-income countries with poor diagnostic and treatment facilities (2). Asian countries constitute a diverse mixture of cultures, geography, ethnicities, and socioeconomic disparities with greatly varied healthcare systems along with large variations in incidence and mortality of breast cancer (2, 4, 11). The majority of Asian countries are developing countries, which does not have robust public health system and healthcare facilities to provide cost-effective diagnostic and therapeutic capacity for patients with cancer and 20% of all the Asian countries have population-based cancer registries (PBCRs), of which only a few countries (e.g., South Korea, Japan, and Singapore) cover the entire population (5). Therefore, due to the lack of representative information, it is pertinent to understand the breast cancer burden using available estimates, which can serve as a valuable tool to develop cancer control policies.

In this study, we examine the geographical disparities in breast cancer mortality at the regional and national levels of East and South Asia, from 1990 to 2019 for women aged 20–84 years. We predicted the breast cancer mortality trends in these regions and countries for the period 2020–2030 using Lee–Carter (LC) model, which accounts for spatial variability and predicts future trends in each country and also allows for mortality risk, longitudinal age trends, and age-adjusted trends over time. To assess the risk-attributable breast cancer mortality in each country under study, we also predicted the mortality trends by risk factors. We conclude by discussing possible underlying risk factors for breast cancer mortality and identifying specific areas of public health concern. To the best of our knowledge, this is the first study using advanced statistical methods to predict breast cancer-related mortality trends by age and risk factors for the next decade at the regional and national levels of East and South Asia.

## Materials and Methods

### Study Population and Data Source

This study used publicly available data from the Global Health Data Exchange (GHDx) (12). As we only used country-specific rates, this study was exempt from ethical review and informed consent according to Code of Federal Regulations [45 CFR 46.101(b)]. This study complies with strengthening the Reporting of Observational Studies in Epidemiology (STROBE) reporting guideline for observational studies.

The GHDx is a health-related database available at the Institute for Health Metrics Evaluation (IHME) and IHME produces estimates of the burden of diseases, including breast cancer. The estimates in this database are created from a repository of input data, which includes data directly obtained from cancer registries, surveillance data, administrative records, and vital registration. It provides a systematic assessment of 369 injuries and diseases, 87 risk factors, and 284 reported reasons of death among 204 countries, twenty-one regions, and seven super-regions. This study covered the female population diagnosed with breast cancer as the per International Classification of Diseases (ICD) version 10 code C50 (11). Countries' corresponding population estimates by age group and calendar year from 1990 to 2019 were obtained from the population estimates 1990–2019 via online GHDx (13).

The GHDx database produced as a result of the global burden of disease 2019 study presents the latest estimates, which are produced via innovative estimation methodologies that have been detailed elsewhere (8, 14, 15). Breast cancer mortality estimates were generated using Cause of Death Ensemble Model (CODEm). The CODEm models the mortality estimates for breast cancer based on all available data and covariates consisting of smoking, education, lagging distribution income, sociodemographic index (SDI), and alcohol consumption (16, 17).

The SDI is a composite indicator of a country's lag-distributed income per capita, average years of schooling among persons above 15 years of age, and total fertility rate (TFR) in females under the age of 25 years (18). The composite SDI is calculated as the geometric mean of these three indices for a given location year. The cutoff values used to determine quintiles for analysis were then calculated using country-level estimates of SDI for the year 2019. This index (SDI) ranges from 0 to 1, i.e., least developed to the most developed ones. The cutoff points for low, low-middle, middle, high-middle, and high SDI countries in 2019 are 0.4547, 0.6076, 0.6895, 0.7905, and 0.8051, respectively.

### Attributable Risk Factors

The comparative risk assessment method has already been integrated into the Global Burden of Disease (GBD) study (19) to measure the burden of multiple causes and impairments of 87 environmental, occupational, metabolic, and behavioral risk factors. Briefly, we chose the mortality component to model the attributable burden of risk factors, following an assessment of the causal evidence in each risk-outcome pair (20).

### Statistical Analysis

In this analysis, death rates (per 100,000) of female breast cancer, corresponding to age groups 20–84 years with 5-year


$$\begin{array}{l} Age\;standardised\;mortality\;rate=\frac{\sum Age\;specific\;rate\times Standard\;population\;in\;corresponding\;age\;group}{\sum Standard\;population} \end{array}$$

age intervals, and successive thirty calendar years from 1990 to 2019, were considered for modeling analysis. LC model (21) was applied to breast cancer death data of each East and South Asian country to project the death rates for the forthcoming 11 years (2020–2030). The model was applied to the two age groups, 20–84 and 50–84 years, by risk factors from 1990 to 2019. The data includes the number of deaths D_x, t_ and exposure to death E_x, t_ for a person of age x last birthday during year t. The central mortality rate m_x, *t*_ is calculated using the formula $m_{x,t}=\frac{D_{x,t}}{E_{x,t}}$. The data to be used are annual age-specific death rates (22).

The LC-based modeling frameworks are viewed in the current literature as among the most efficient and transparent methods of modeling and projecting mortality improvements. Previously, scholars have used this approach to forecast all-cause and cause-specific mortality in different countries (22–26). The general expression of the model is expressed as: $m_{x,t}=e^{a_{x}+b_{x}+k_{t}+\varepsilon_{x,t}}$, where, *m*_*x, t*_ is the central mortality rate at age x and year t, and *a*_x_ is the average (over time) log-mortality at age x, and b_x_ measures the response at age x to changes in the overall level of mortality over time, and *k*_t_ represents the overall level of mortality in year t, and ε_x, *t*_ is the residual term. The Singular Value Decomposition (SVD) algorithm was used to forecast the general mortality index for the period 2020 to 2030, for each country separately.

Based on age-standardized mortality estimates of 2019, we ranked the risk factors of breast cancer for East and South Asian countries. The four risk factors that were ranked first were selected for further prediction analysis (Supplementary Figure 1). The spatial/regional disparity in mortality trends attributable to high body mass index (BMI), high fasting plasma glucose (FPG), alcohol use, and diet high in red meat were predicted from 1990 to 2030 among the female population of age 50–84 years. In addition, the autoregressive integrated moving average (ARIMA) method (27, 28) was employed to predict the SDI values from 1990 to 2030 for each country.

The mortality modeling methodology was implemented using publically available R programming functions with package ilc (29), which facilitates both the preparation of mortality data, fitting, and analysis of the given log-linear modeling structures. The package also incorporates methods to produce future mortality rates forecasts and compute the corresponding future life expectancy. Further, the forecast package in R (30) was used to predict the future SDI values for each country.

This study adopted the following steps of analysis. First, we predicted the age-specific mortality rates for 11 years from 2020 to 2030 and then the change between 2019 and 2030 in estimated age-specific mortality rates was expressed as a percentage (the difference between morality rates at two points divided by the initial mortality value and multiplied by 100). Age-standardized mortality rates were computed using the direct method based on the WHO World Standard Population (31). The direct calculation of the age-standardized mortality rate was as follows (32):

We also predicted risk attributable mortality rates by potential breast cancer risk factors among females of aged 50–84 years. Finally, we projected the SDI value for the same prediction period and assessed the association between SDI and mortality changes using the spearman correlation coefficient test during the entire period from 1990 to 2030. Additionally, to test the sensitivity of the method, we evaluated the performance of the LC model in comparison with an alternative stochastic mortality model called Booth–Maindonald–Smith (BMS) model (33) and predicted the breast cancer mortality trends by using both the models. A detailed description of about BMS method has been provided elsewhere (22, 33).

## Results

### Fitting of the Model to Breast Cancer Mortality

The results of estimation of parameters in the LC model, for twelve locations (2 regions and 10 countries) are described in Supplementary Figure 2. The estimated values of age-dependent parameters α_x_ and β_x_, and the time-dependent parameter k_t_ are reported for each country. Applying SVD to the matrix M_x, t_, we obtained an explained variance of 83.2, 82.8, 81.7, 90.9, 94, 73.9, 85.5, 74.5, 96.8, 94.5, 89, and 76% by fitted LC model for East Asia, China, Japan, North Korea, South Korea, Mongolia, South Asia, India, Pakistan, Afghanistan, Nepal, and Bangladesh, respectively. These results indicate that the LC model fitted breast cancer-related mortality data reasonably well. Further, the forecasted breast cancer death rates from 2020 to 2030 had low values of mean squared error (MSE), Akaike's Information Criteria (AIC), and Bayesian Information Criteria (BIC).

### Trends and Changes in Age-Standardized Breast Cancer Death Rates From 1990 to 2030

**Figure 1:**
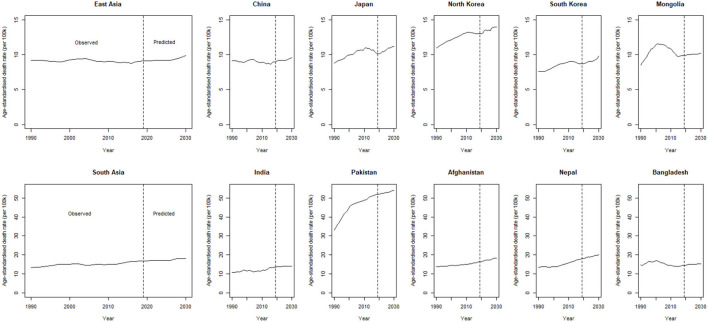
Trends in observed and predicted age-standardized death rate (ASDR) (per 100,000) of breast cancer, from 1990 to 2030 at a regional and national level of East and South Asia.

The trends in observed and predicted age-standardized death rates (ASDRs) (per 100,000) of female breast cancer at regional and national levels from 1990 to 2030 are depicted in Figure 1. Overall ASDR of breast cancer is predicted to increase by 7.0% from 9.20 (95% CI: 6.04–12.12) per 100,000 in 1990 to 9.88 (95% CI: 7.12–11.4) per 100,000 in 2030 in East Asia, and by about 35% from 13.4 (95% CI: 9.21–16.02) per 100,000 in 1990 to 18.1 (95% CI: 13.23–21.10) per 100,000 in 2030 in South Asia. At the national level, the highest percent change in ASDR between 1990 and 2030 is predicted in Pakistan (a 62% increase) and Nepal (a 47% increase) (Figure 1, Supplementary Table 1).

**Figure 2:**
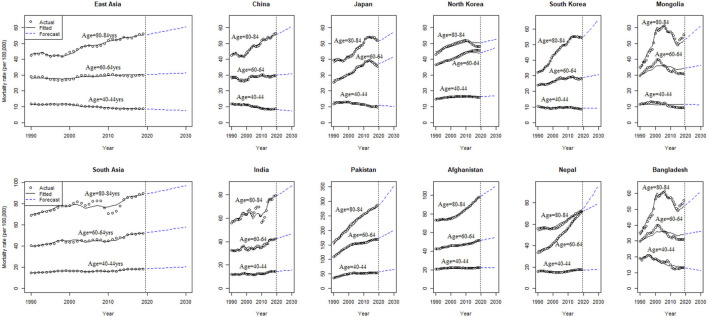
Age-specific observed, estimated, and predicted breast cancer mortality rates (per 100k) from 1990 to 2030, at the regional and national level of East and South Asia.

The age-specific breast cancer mortality rates (per 100,000) in East and South Asian regions from 1990 to 2030 are shown in Figure 2. Mortality rates are predicted to increase with age, particularly for the age group 80–84 years in all the East and South Asian regions. A significantly higher percent change in breast cancer mortality between 2019 and 2030 for females of age group 75–79 years is observed in South Korea [21.0 (95% CI: 10.9–71.2)] and North Korea [9.4 (6.9–61.8)] among East Asian regions. The predicted breast cancer mortality rates are greater in South Asian regions than East Asian and significantly higher mortality percent change between 2019 and 2030 is predicted in Pakistan [21.6 (20.6–94.7)], Afghanistan [13.3 (4.0–80.8)], and Nepal [36.6 (11.1–125.7)] for age group 80–84 years (Table 1).

**Table 1:** Estimated age-specific mortality rates (per 100 k), percent change between 2019 and 2030, for breast cancer, in East and South Asian regions.

	**2019**	**2030**	
	**Mortality rate (95% CI)**	**Mortality rate (95% CI)**	**%Change, between 2019 and 2030**
**East Asia**			
Age 20–24	0.2 (0.2, 0.3)	0.1 (0.1, 0.2)	−32.9 (−66.9, 39.4)
25–29	0.7 (0.5, 0.9)	0.5 (0.3, 0.7)	−27.5 (−62.6, 43.8)
30–34	2.3 (1.8, 3)	1.6 (1.1, 2.5)	−30 (−64.1, 39.7)
35–39	5.2 (4, 6.6)	4.1 (3.1, 5.6)	−20.6 (−53.3, 38.4)
40–44	8.9 (7, 11.1)	7.5 (5.8, 9.8)	−15.4 (−47.6, 38.5)
45–49	12.4 (9.8, 15.5)	9.7 (6.4, 14.5)	−21.8 (−58.5, 48.4)
50–54	19.7 (15.6, 24.5)	18.7 (16.3, 21.4)	−5.2 (−33.4, 37.4)
55–59	26.7 (21.4, 33.4)	29.2 (27.2, 31.3)	9.1 (−6.3, 27.1)
60–64	30 (24.2, 36.8)	32.1 (29.4, 35.1)	6.9 (−4.6, 21.4)
65–69	35 (28.6, 42.6)	37.7 (32.9, 43.2)	7.7 (1.3, 15)
70–74	41.2 (33.5, 50.1)	43.9 (37.6, 51.3)	6.5 (2.3, 12.2)
75–79	50.1 (41.4, 60.3)	55.2 (44, 69.2)	10.1 (6.3, 14.7)
80–84	56 (45.4, 66.8)	61.1 (49.5, 75.4)	9 (8.1, 13)
**China**			
Age 20–24	0.2 (0.1, 0.3)	0.1 (0.1, 0.2)	−33.9 (−72.8, 65.4)
25–29	0.7 (0.5, 0.9)	0.5 (0.3, 0.8)	−28.2 (−68.3, 66.6)
30–34	2.3 (1.7, 2.9)	1.6 (0.9, 2.8)	−31.1 (−69.7, 61.7)
35–39	5.1 (3.9, 6.5)	4 (2.7, 6)	−21.4 (−58.8, 55.7)
40–44	8.7 (6.7, 11)	7.3 (5.1, 10.4)	−16.2 (−53.5, 54.4)
45–49	12 (9.3, 15.2)	9.1 (5.2, 16.1)	−23.5 (−66, 73.5)
50–54	19.3 (15.1, 24.2)	18.1 (14.8, 22)	−6.4 (−38.8, 45.8)
55–59	26.2 (20.6, 33.3)	28.7 (26.2, 31.5)	9.4 (−5.3, 26.8)
60–64	29.6 (23.5, 36.7)	31.7 (28.2, 35.6)	7.1 (−3.1, 19.8)
65–69	34.8 (28.1, 42.8)	37.2 (31.2, 44.3)	7.0 (3.7, 11.0)
70–74	41.1 (33.3, 50.3)	43.6 (35.6, 53.3)	6.0 (5.9, 7.2)
75–79	50.3 (41.2, 61)	55.3 (40.9, 74.7)	10 (−0.7, 22.5)
80–84	56.3 (45.3, 67.6)	61.4 (46.3, 81.4)	9.1 (2.3, 20.5)
**Japan**			
Age 20–24	0.1 (0.1, 0.1)	0.1 (0.1, 0.1)	10.2 (4.8, 16.1)
25–29	0.6 (0.5, 0.6)	0.6 (0.6, 0.6)	4.2 (0, 8.1)
30–34	2.1 (1.9, 2.2)	2.1 (1.9, 2.3)	2.1 (−0.8, 4.1)
35–39	4.9 (4.6, 5.3)	5.1 (4.6, 5.6)	3.6 (−0.5, 5.7)
40–44	9.8 (9.3, 10.4)	10.2 (9.6, 10.9)	4.9 (3.5, 5.3)
45–49	16.6 (15.7, 17.5)	17.9 (17.4, 18.5)	8.3 (5.7, 10.6)
50–54	25.5 (24.2, 26.9)	28.5 (28, 29.1)	12 (3.8, 20.1)
55–59	32.2 (30.4, 34.2)	37.3 (34.9, 39.8)	15.8 (2.1, 30.9)
60–64	35.5 (33.3, 37.8)	40.3 (36.4, 44.6)	13.3 (−3.7, 34)
65–69	37.8 (34.6, 40.3)	41 (37.2, 45.2)	8.6 (−7.6, 30.9)
70–74	39.2 (34.6, 42.5)	42.3 (38.6, 46.4)	7.9 (−9.1, 34.3)
75–79	46.4 (38.4, 51.2)	50.2 (45.3, 55.7)	8.3 (−11.5, 45.2)
80–84	52.4 (38, 60.4)	55.6 (50.5, 61.3)	6.2 (−16.5, 61.5)
**North Korea**			
Age 20–24	0.3 (0.2, 0.5)	0.3 (0.3, 0.3)	−0.7 (−44.1, 86.4)
25–29	1.1 (0.6, 2)	1.1 (1.1, 1.1)	−0.1 (−44.9, 101.2)
30–34	3.7 (1.8, 6.5)	3.7 (3.7, 3.7)	−1 (−44.2, 98.8)
35–39	8.9 (4.6, 15.7)	9.2 (9.1, 9.3)	3.4 (−41.8, 102)
40–44	16 (8.8, 26.5)	16.9 (16.6, 17.1)	5.1 (−37.3, 94.5)
45–49	26.9 (15.9, 42.9)	28.5 (27.8, 29.3)	5.9 (−35.2, 84.6)
50–54	35.7 (21.2, 55.1)	37.8 (36.6, 39)	6 (−33.5, 84)
55–59	44.8 (28.1, 69.4)	46.7 (44.9, 48.5)	4.1 (−35.3, 72.4)
60–64	45.4 (29.2, 67.1)	47.1 (45.4, 48.9)	3.8 (2.4, 67.5)
65–69	45.4 (29.8, 65.6)	48.1 (46.3, 50)	5.9 (4.4, 67.5)
70–74	43.7 (30.8, 61.2)	47.2 (45.7, 48.7)	8 (5.4, 58)
75–79	46.5 (32.3, 65)	50.9 (49.5, 52.3)	9.4 (6.9, 61.8)
80–84	48.1 (32.5, 68.8)	52.4 (51.3, 53.5)	8.9 (2.4, 64.9)
**South Korea**			
Age 20–24	0.1 (0.1, 0.1)	0.1 (0.1, 0.1)	−14 (−36.7, 21.5)
25–29	0.5 (0.3, 0.7)	0.4 (0.4, 0.5)	−14 (−35.2, 14.9)
30–34	2.2 (1.6, 2.9)	2.1 (1.9, 2.2)	−5.7 (−23.5, 20.1)
35–39	5.1 (3.8, 6.6)	5.6 (5.6, 5.6)	10.9 (−14.4, 46.1)
40–44	8.7 (6.7, 10.8)	9.2 (9.2, 9.3)	6.3 (−13.6, 36.8)
45–49	13.6 (10.9, 16.6)	13.8 (13.6, 14)	1.7 (−15.8, 25.1)
50–54	18.2 (14.5, 21.9)	18.5 (18.4, 18.7)	2.1 (−14.5, 26.4)
55–59	24.2 (19.6, 29.1)	25.6 (25.2, 26.1)	5.8 (−13.4, 32.8)
60–64	28.1 (22.7, 34.3)	30.5 (29.5, 31.4)	8.3 (−13.9, 38.5)
65–69	33.6 (27.2, 41.1)	36.6 (34.8, 38.5)	8.9 (5.2, 41.3)
70–74	38.7 (31.1, 47.3)	43.5 (40.8, 46.4)	12.4 (10.6, 49.2)
75–79	48 (37.5, 58.9)	58 (52.5, 64.2)	21 (10.9, 71.2)
80–84	54.6 (39.9, 71.3)	63.7 (58.2, 69.7)	16.6 (13.3, 75)
**Mongolia**			
Age 20–24	0.2 (0.1, 0.4)	0.3 (0.3, 0.3)	10.5 (−37.3, 111)
25–29	0.6 (0.3, 1)	0.7 (0.6, 0.9)	20.1 (−36.4, 146.5)
30–34	2.8 (1.6, 4.6)	3.3 (2.8, 3.8)	15 (−38.5, 129.1)
35–39	5.8 (3.6, 8.9)	6.8 (6.1, 7.6)	16.2 (−32.2, 111.9)
40–44	9.5 (5.8, 14.2)	11.9 (10.6, 13.4)	25.4 (−25.7, 130.4)
45–49	15.6 (9.9, 23.8)	19.4 (16.6, 22.6)	24.4 (−30.2, 128.4)
50–54	21.8 (13.8, 32.5)	26.6 (23.1, 30.7)	21.8 (−29, 122.1)
55–59	27.3 (17.5, 39.8)	32.7 (28, 38.2)	19.6 (−29.7, 118.6)
60–64	30.9 (20.7, 45.3)	35.6 (31.7, 40)	15.2 (−30.1, 93.8)
65–69	38.1 (25.4, 56.2)	41.1 (36.7, 46.1)	8 (−34.8, 81.9)
70–74	43.2 (29.2, 61.6)	43 (40.5, 45.7)	−0.3 (−34.2, 56.7)
75–79	48.5 (32.6, 68.5)	49.2 (43, 56.2)	1.4 (−37.1, 72.5)
80–84	55.6 (36.8, 81.1)	53.5 (47, 61)	−3.7 (−42.1, 66)
**South Asia**			
Age 20–24	1 (0.8, 1.3)	1.2 (1.1, 1.4)	18.8 (−17, 77.3)
25–29	2.4 (1.9, 3)	2.9 (2.6, 3.2)	18.8 (−14.7, 68.5)
30–34	4.7 (3.7, 5.8)	5.1 (4.8, 5.5)	8.8 (−17.3, 46.8)
35–39	9.3 (7.5, 11.6)	10.2 (9.5, 10.8)	8.9 (−18.1, 45)
40–44	18.4 (14.5, 22.4)	20.5 (19, 22.1)	11.3 (−15.3, 52.1)
45–49	26.9 (21.7, 32.9)	29.9 (28.2, 31.7)	11.3 (−14.1, 45.8)
50–54	45.9 (35.3, 57.3)	53.6 (47.9, 59.8)	16.6 (−16.4, 69.5)
55–59	50.5 (40.2, 61.6)	58.2 (53, 64)	15.4 (14, 59.1)
60–64	52.2 (42.9, 62.4)	58.8 (53.7, 64.3)	12.5 (−14, 49.8)
65–69	58.7 (48.1, 69.5)	64.1 (59.6, 68.9)	9.1 (4.3, 43.2)
70–74	70.1 (57.6, 83.5)	75.5 (70.6, 80.6)	7.7 (−15.5, 40)
75–79	75.3 (61.8, 89.2)	75.8 (73.6, 78.1)	0.7 (−17.5, 26.4)
80–84	89.7 (71.3, 108.1)	96.6 (89.8, 103.9)	7.7 (5.9, 45.7)
**India**			
Age 20–24	0.6 (0.5, 0.8)	0.6 (0.6, 0.6)	−1.4 (−27.6, 39)
25–29	1.6 (1.2, 2.1)	1.7 (1.5, 1.9)	6.7 (−25.8, 58.2)
30–34	3.7 (2.8, 4.8)	3.7 (3.5, 3.8)	−0.2 (−26.5, 38)
35–39	8.2 (6.3, 10.6)	8.6 (8.1, 9.2)	5 (−23.4, 47)
40–44	14.4 (10.8, 18.7)	16 (14.4, 17.7)	11.5 (−22.7, 64.5)
45–49	20.5 (15.7, 26.3)	22.6 (20.9, 24.3)	10.3 (−20.4, 55.1)
50–54	36.7 (25.5, 49.2)	47 (37.9, 58.3)	28.1 (−23.1, 128.2)
55–59	40.3 (29.5, 53.1)	46.6 (40.6, 53.5)	15.7 (3.5, 81.4)
60–64	42.4 (32.5, 54.1)	47.6 (42, 53.9)	12.2 (9.4, 65.9)
65–69	48 (38.2, 60.3)	51.7 (47.3, 56.5)	7.7 (5.7, 47.9)
70–74	57.4 (44.9, 72.6)	60.9 (56.1, 66.1)	6.2 (4.7, 47.2)
75–79	64.2 (50.2, 80.5)	64.9 (62.1, 67.9)	1.1 (−22.9, 35.2)
80–84	79.3 (59.5, 102)	87.9 (77, 100.2)	10.8 (2.5, 68.3)
**Pakistan**			
Age 20–24	3.8 (2.4, 5.7)	4.9 (4.5, 5.3)	28 (−21.1, 121.4)
25–29	8.7 (5.7, 12.4)	10.9 (10.2, 11.6)	25 (−17.6, 102.2)
30–34	14.1 (9.2, 20.6)	17.1 (16.2, 18.2)	21.4 (20.6, 98.1)
35–39	21.7 (14.8, 32.5)	25.6 (24.4, 26.8)	17.8 (12.9, 81.7)
40–44	54 (35.7, 80.2)	66.1 (63.1, 69.2)	22.3 (−21.4, 94.1)
45–49	83 (56.7, 122.7)	98.6 (95.4, 101.9)	18.8 (10.3, 79.7)
50–54	123.4 (85.9, 175.2)	146.3 (140.8, 151.9)	18.5 (14.6, 76.7)
55–59	151 (106.1, 214.9)	181.2 (173.2, 189.5)	20 (19.4, 78.6)
60–64	169 (118.3, 232.5)	200.8 (191.1, 211.1)	18.8 (17.8, 78.4)
65–69	194 (137.8, 269.2)	231.9 (219.8, 244.6)	19.5 (18.4, 77.5)
70–74	238.5 (168.4, 327.3)	284.3 (267.8, 301.7)	19.2 (18.2, 79.2)
75–79	251.7 (172.8, 352.9)	298.6 (281.3, 316.9)	18.6 (17.3, 83.3)
80–84	286.3 (191.8, 419.6)	348.3 (324.8, 373.5)	21.6 (20.6, 94.7)
**Afghanistan**			
Age 20–24	0.9 (0.5, 1.4)	0.8 (0.8, 0.8)	−4.4 (−40.4, 62.5)
25–29	2.1 (1.2, 3.5)	2 (2, 2)	−7.4 (−42.5, 60.3)
30–34	5.9 (3.4, 9.6)	5.4 (5.4, 5.5)	−7.8 (−42.9, 57.4)
35–39	11.6 (7.2, 18.1)	11.3 (11.2, 11.3)	−3.1 (−37.8, 56.4)
40–44	22.2 (13.9, 32.8)	22.5 (22.5, 22.6)	1.6 (−31.5, 63.2)
45–49	27.2 (17, 40.8)	27 (26.9, 27.2)	−0.6 (−34, 59.5)
50–54	33.2 (20.7, 50)	34.2 (33.8, 34.6)	3.1 (2.4, 66.8)
55–59	47.1 (30.3, 68.6)	49.7 (48.8, 50.5)	5.5 (4.9, 66.7)
60–64	51.7 (33.2, 77.2)	55.1 (54, 56.1)	6.5 (3.2, 69.0)
65–69	51.2 (33.3, 72.9)	56.6 (55.1, 58.1)	10.5 (4.4, 74.5)
70–74	59.2 (39.7, 86.6)	65.8 (63.8, 67.8)	11.1 (6.3, 70.9)
75–79	84 (55.4, 120.2)	94.9 (91.7, 98.2)	12.9 (7.7, 77.2)
80–84	98.1 (63.7, 143)	111.2 (107.3, 115.2)	13.3 (4.0, 80.8)
**Nepal**			
Age 20–24	0.2 (0.1, 0.3)	0.2 (0.2, 0.2)	6 (−34.9, 91.3)
25–29	0.6 (0.3, 1)	0.6 (0.6, 0.6)	−7.4 (−41.5, 67.5)
30–34	2.2 (1.2, 3.5)	2.2 (2.2, 2.2)	0.6 (−38, 89.6)
35–39	6.3 (3.7, 9.8)	6.3 (6.2, 6.3)	−0.5 (−37.2, 70.8)
40–44	17.8 (10.9, 27.1)	17.8 (17.6, 18)	0 (−35, 65.1)
45–49	24.4 (15.2, 38.1)	22.6 (22.4, 22.8)	−7.2 (−41, 50.5)
50–54	45.6 (28.6, 68.1)	45.6 (44.9, 46.4)	0.2 (−34.1, 62.3)
55–59	87.8 (57.8, 126.9)	101.1 (98.8, 103.5)	15.2 (12.1, 78.9)
60–64	72.9 (47.6, 103.7)	79.6 (77.5, 81.7)	9.2 (6.3, 71.8)
65–69	70.2 (45.8, 103.6)	79.1 (76.5, 81.8)	12.6 (10.2, 78.7)
70–74	64.5 (43.7, 95.1)	76.5 (73.3, 79.8)	18.6 (14, 82.4)
75–79	68.1 (45.1, 96.6)	85 (80.5, 89.8)	24.9 (16.7, 99.1)
80–84	71.5 (46.4, 102.4)	97.6 (91.1, 104.7)	36.6 (11.1, 125.7)
**Bangladesh**			
Age 20–24	0.9 (0.5, 1.4)	1 (0.6, 1.7)	14.6 (−57.3, 223.4)
25–29	2 (1.2, 3.1)	2.6 (1.1, 5.9)	29.2 (−64.9, 386.3)
30–34	3 (1.8, 4.5)	4.7 (1.3, 16.1)	55.5 (−70.5, 795.6)
35–39	5.6 (3.5, 8.2)	8.2 (2.7, 25)	48.2 (−67.1, 616.5)
40–44	13.3 (8.6, 19.2)	19 (6.4, 56.3)	43 (−66.7, 551.7)
45–49	22.4 (15.2, 32)	28.6 (15.3, 53.5)	27.9 (−52.2, 251.5)
50–54	51.7 (35.5, 73.1)	56.5 (40.1, 79.7)	9.3 (−45.1, 124.6)
55–59	44.9 (30.9, 61.7)	49 (34.3, 69.9)	9 (−44.4, 126.3)
60–64	41.7 (29.4, 59.2)	53.9 (26.2, 110.7)	29.1 (−55.7, 276)
65–69	53.7 (37.1, 75.6)	51.2 (52.9, 49.6)	−4.6 (−30, 33.6)
70–74	68.2 (48.2, 93.5)	62 (73.4, 52.4)	−9.1 (−21.5, 8.8)
75–79	57.9 (39.5, 83.9)	53.9 (53.6, 54.2)	−6.9 (−36.1, 37.1)
80–84	65.9 (43.9, 95.3)	53.2 (90, 31.4)	−19.3 (−5.6, −28.4)

### Breast Cancer Mortality and Attributable Risk Factors

**Figure 3:**
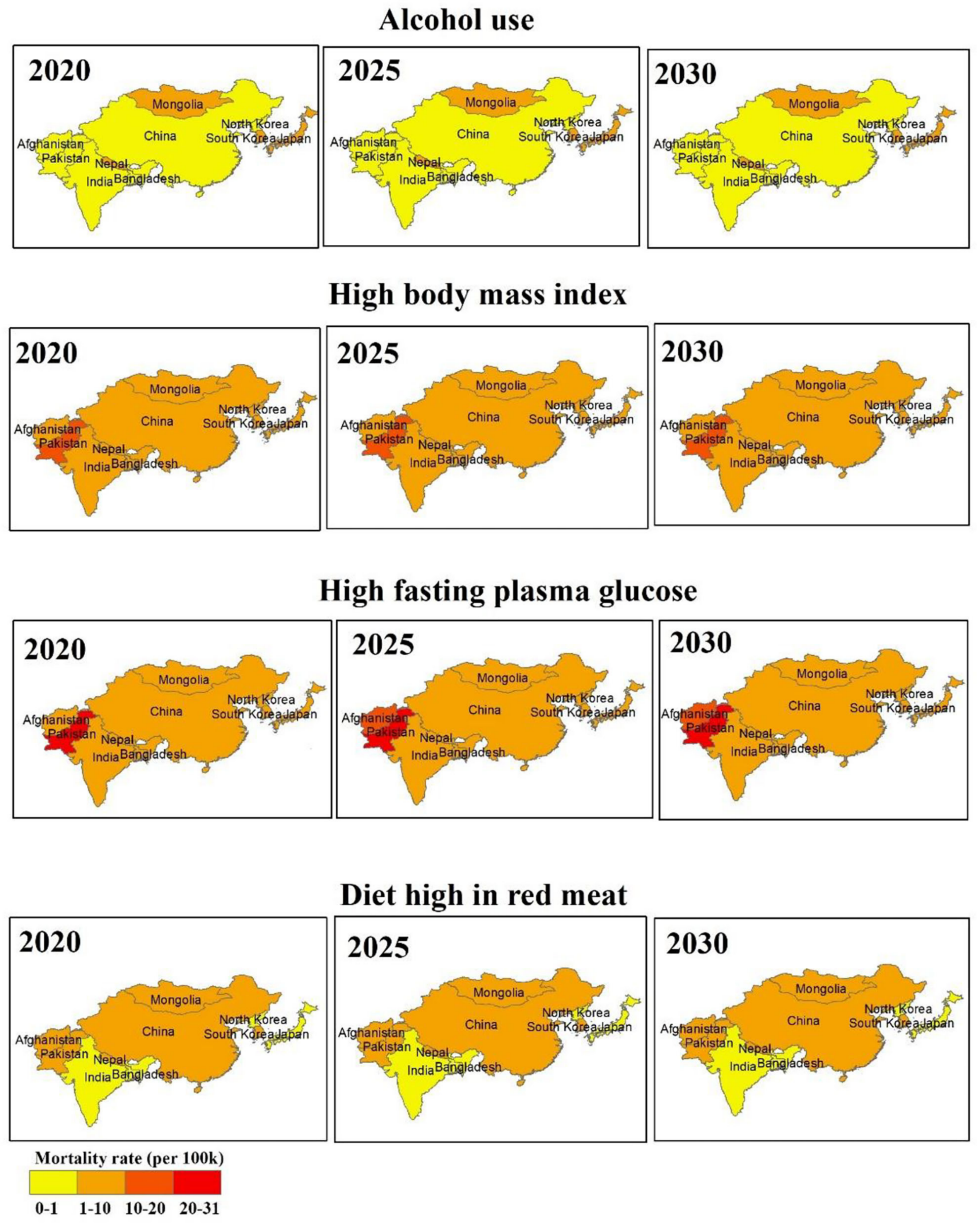
Regional disparity of mean predicted risk attributable mortality rates (per 100 k) for the female of aged 50–84 years old, for the year 2020, 2025, and 2030.

**Figure 4:**
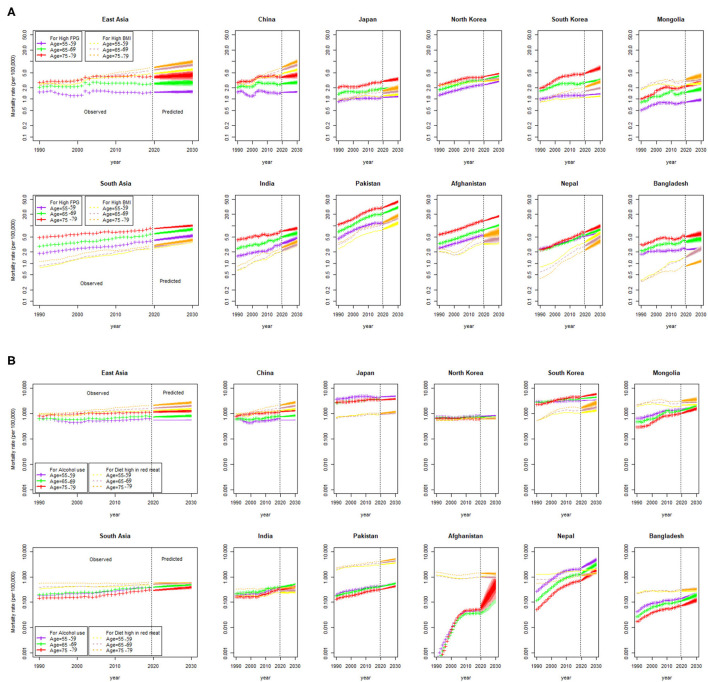
Age-specific risk attributable breast cancer mortality rate (per 100,000) from 1990 to 2030, at a regional and national level. Shades in the fan represent prediction intervals at 80% and 90% level, **(A)** Predicted mortality rates attributable to high fasting plasma glucose (FPG) and high body mass index (BMI), and **(B)** Predicted mortality rates attributable to alcohol use and for a diet high in red meat.

The high BMI- and high FPG-related mortality rates (per 100,000) are increasing in both the regions, with the highest increase observed in Pakistan and Afghanistan during 2020–2030. The alcohol association to increase mortality risk is expected in Japan, Mongolia, South Korea, and Nepal during the next decade than other countries. Six out of ten countries (China, Mongolia, South Korea, Pakistan, Afghanistan, and Nepal) are predicted to have high mortality attributable to a diet high in red meat during 2020–2030 (Figure 3). The age-specific mortality rates (per 100,000) by different risk factors are projected from 2020 to 2030 and are presented in Figure 4. Overall increasing mortality trend is predicted from 2020 to 2030 for the females in the 75-plus age groups who had high FPG and high BMI. The risk-attributable mortality rates are higher in South Asian countries when compared with the East Asian countries.

Additionally, we also reported life expectancy trends of patients with breast cancer of age 50–84 years (all-cause) (Supplementary Figure 3). We observed that overall in East Asia, China, and South Korea, the life expectancy of a female patient with breast cancer might increase during the prediction period 2020–2030; whereas for other locations, it is expected to decrease or remain constant during the same period (Supplementary Figure 3).

### Trend Prediction in SDI and Its Association With Reduced Mortality Risk

**Figure 5:**
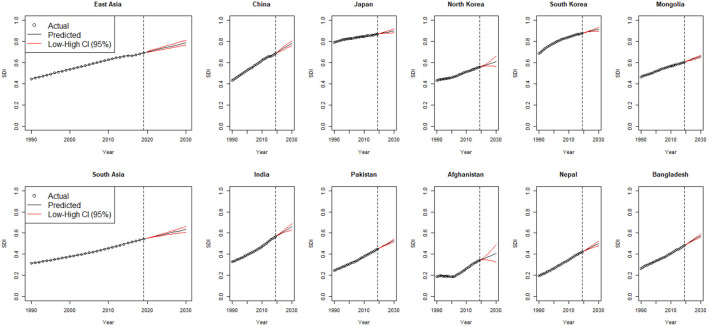
Observed, estimated, and predicted trends in SDI from 1990 to 2030, by region and countries; SDI, sociodemographic index; CI, confidence interval.

The country- and region-wise trends in SDI for 1990 to 2030 are given in Figure 5. We observe an increasing SDI trend during the entire study duration among all the Asian regions and countries, including East and South Asia, with comparatively high SDI predicted in Japan and South Korea.

**Figure 6:**
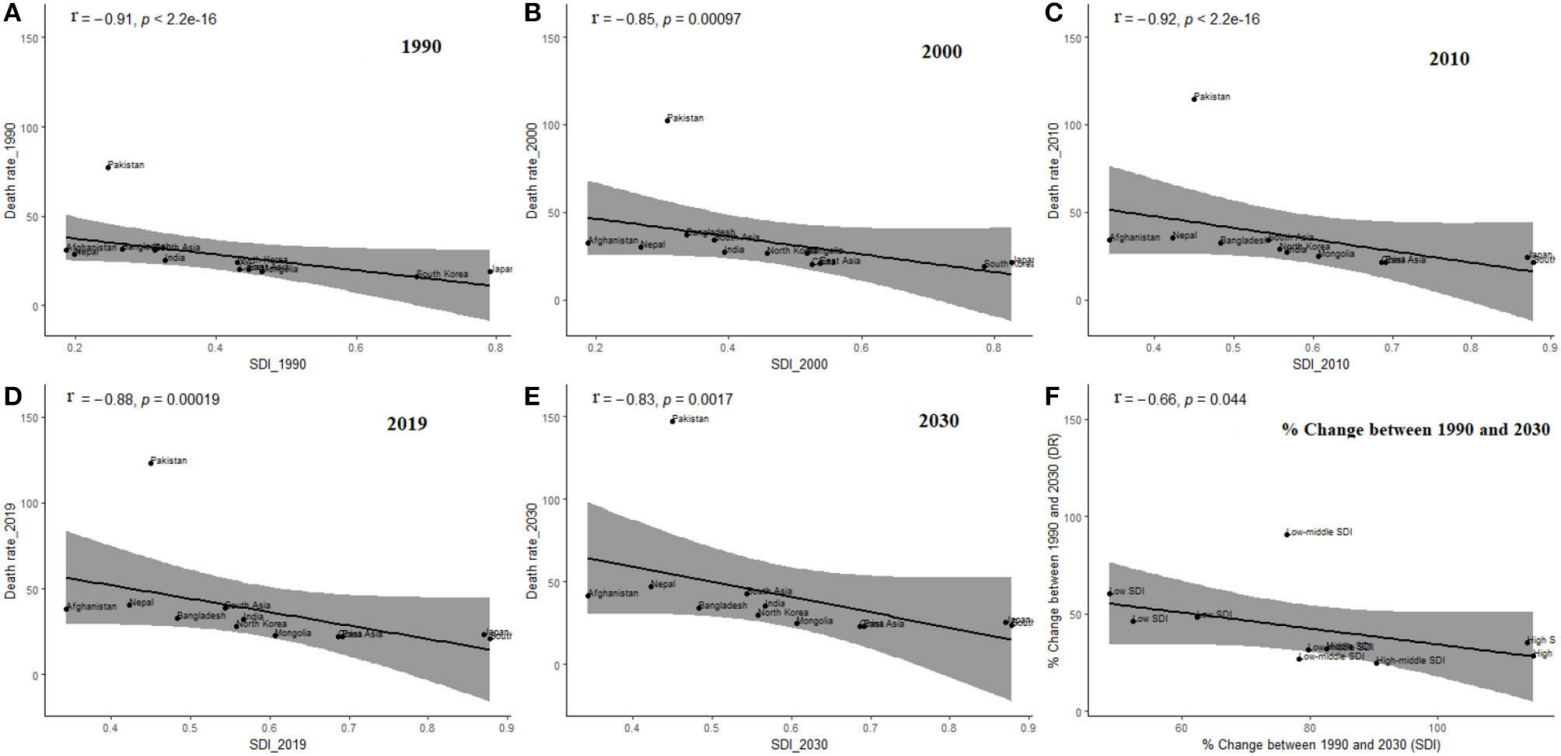
Relationship between death rate (DR) and sociodemographic index (SDI) within Asian region including East and South Asia, in different prediction period, **(A)** 1990, **(B)** 2000, **(C)** 2010, **(D)** 2019, **(E)** 2030, **(F)** % Change between 1990 and 2030; The black dots represent the breast cancer related DR of each country, the black lines are the fitting lines of correlation trend, and grey shaded area is the 95% confidence interval for fitting results. DR, mean death rate (per 100k) from age 20 to 84 years.

Figure 6 presents the association between SDI and mortality rates from 1990 to 2030. A significantly high negative correlation (*r* ≥ 0.83, *p* ≤ 0.001) was observed between SDI and mortality rate across all the countries in both the prediction and forecasted periods (1990, 2000, 2010, 2020, and 2030). Figure 6F shows the association between percent change in SDI between 1990 and 2030 and percent change in mortality rates in East and South Asian countries. Decreases in percent change in mortality rates did occur in many locations with increases in percent change in SDI, notably, for locations belonging to the high SDI quintiles (Japan and South Korea). The largest increases in mortality rate are expected to occur in low-middle-SDI and low-SDI countries led by Pakistan.

As a sensitivity check, we estimated the mortality change using both models (LC and BMS; Supplementary Figure 4) and evaluated the model's performance by explaining variance (%) (Supplementary Table 2) and error measures (Supplementary Table 3) including MSE. We predicted the death rates from 2020 to 2030 using both the models (Supplementary Figure 5) and almost similar results were obtained by these methods.

## Discussion

This study estimated the future trends of breast cancer-related mortality in East and South Asian countries. We also predicted the future SDI trends of regions and countries from 1990 to 2030 and analyzed the relation between SDI and breast cancer mortality risk. There was an inverse association between SDI and mortality rates of breast cancer within East and South Asian countries. The largest percent increase in mortality rate occurred in low-middle-SDI and low-SDI countries led by Pakistan. These findings suggest the contribution of socioeconomic development in reduced risk of breast cancer mortality.

Sociodemographic factors, lack of beliefs in preventive health, health literacy, cultural influences, socioeconomic status as well as lack of access to healthcare are the challenges to breast cancer management and care in the countries under this study (34–37). Breast cancer mortality is predicted to increase continuously; however, if effective control and prevention are not implemented, mortality is expected to continue to surge even more in the coming decades because of substantial demographic changes, which may create considerable public health challenges for these Asian countries. Previous studies have also found that breast cancer-related mortality has been increasing more rapidly in Asia than the global average (38, 39). These increasing trends have many possible reasons apart from demographic changes, which includes risk factors such as smoking, alcohol consumption, physical inactivity, low fiber and high-fat diet, obesity (40, 41), ambient air pollution, and air pollution due to the burning of solid fuels in houses (42).

Breast cancer treatment depends upon molecular subtypes which are generally characterized on the basis of the status of estrogen receptor (ER), progesterone receptor (PR) and human epidermal growth factor receptor 2 (HER2), breast cancer which is negative in ER, PR, and HER2 is termed as triple-negative breast cancer (TNBC) (43). TNBC is generally more aggressive and has a poor prognosis (44). Previous studies have shown that patients with TNBC in Pakistan, China, and India are generally of younger age and present with large invasive tumors, high node positivity, and high histological grade (45–47). Late-disease presentation is another common issue in Asian patients with breast cancer, which results in poor prognosis (48). Among cultural factors, in several Asian countries, females tend to hide tumor formation on breast from family and husband, which also delays the diagnosis leading to poor prognoses (1).

The mortality trend from breast cancer is predicted to increase in the next decade compared with the past decades in both East and South Asian countries. Japan officially recommended in 2004 that women should start mammographic screening at the age of 40 years, and once biannually after that (49). However, there is no clear guideline for the termination of the age of screening, and elderly women do not pay enough attention to breast screening and self-examination, which also contributes to the high mortality rate. Clinical breast examinations, as well as mammograms, are widely available in public primary health care institutions in Japan, which may also account for the high incidence rates of breast cancer along with lower mortality rates and higher 5-year survival rates in Japan (49).

The South Asian region demonstrated increasing mortality trends for all its countries with Pakistan, India, Nepal, and Afghanistan demonstrating a steady upward trend in mortality in the next decade. The population aging is a non-modifiable risk factor for developing breast cancer leading to mortality in Asian women (50–52). Correspondingly, this study shows that mortality is predicted to increase with age, particularly among 80–84 years old in all the East and South Asian countries. Previous studies have also identified mortality risk increases with age with a peak of breast cancer-related mortality at approximately 85 years (1, 4, 24, 53).

Most of the developed and developing countries of Asia are increasingly adopting a Westernized lifestyle, such as a diet high in fat and processed meat, low in fruits and vegetables, and lack of (or low) physical activity leading to obesity, which is linked with breast cancer apart from several other health risks of obesity (54). On average, obesity increases breast cancer-related mortality risk by ~30–40% (55–58). In this study, breast cancer mortality trends related to high BMI, high FPG, alcohol use, and a diet high in red meat are also predicted for the period 2020–2030 among females of age 50–84 years. The high BMI- and high FPG-related breast cancer mortality rates were high in both the Asian regions and country-wise, these were the highest in Pakistan and Afghanistan, whereas alcohol-related mortality rate was highest in Japan, Mongolia, South Korea, and Nepal.

Among 10 countries, six countries (China, Mongolia, South Korea, Pakistan, Afghanistan, and Nepal) have shown a high-predicted mortality rate attributable to a diet high in red meat. Another study has shown that higher red meat consumption, especially processed red meat, is associated with an increased risk of breast cancer-related mortality, which has a relationship between red meat consumption and mortality risk (59). A meta-analysis of prospective studies investigated the association between red meat intake and risk of breast cancer and has shown that the pooled relative risk of breast cancer for the highest vs. the lowest categories of red meat intake were 1.10 (95% CI, 1.02–1.16) and each additional intake of 120 g of red meat per day was associated with 11% additional risk of breast cancer (59). The potential biological mechanisms include heme iron and non-heme iron pro-oxidant activity and the carcinogenic by-products compounds produced in the high-temperature cooking of red meat (60). The consumption of red meat has increased tremendously in the East Asian countries in the last few decades and is expected to be the region with one of the highest red meat consumption by 2030 (61).

This study predicted life expectancy trends among patients with breast cancer of age 50–84 years. We observed that the life expectancy of a female patient with breast cancer in East Asia, China, and South Korea, might increase while for other countries, it is expected to decrease or remain constant during the prediction period 2020–2030. Increases in life expectancy might be due to changing lifestyles, provision of better hospitalization, and fulfillment of required nutritious standards in these countries (62). In addition, population-based breast cancer screening programs are also available in some of the East Asian countries, include China and South Korea (63–66).

Our research results are estimated by integrating data from multiple sources in the GBD network using a constantly updated modeling process; there are, however, few limitations in this study. First, although GBD estimates of the breast cancer burden are based on multiple sources of data, due to lack of quality PBCRs in low-resource countries of East and South Asia, the estimates are produced using spatiotemporal modeling, because of which, these estimates may be biased. Moreover, the uncertainty intervals may be too wide for a few countries. Second, we have described only the overall trends for each country, but did not describe the subadministrative regions of each country. Third, the estimates may also be biased because of the lack of diagnostic facilities, or healthcare workers expertise in proper stage and grade detection of tumors. Therefore, these estimates cannot be considered a substitute for data collection using quality PBCRs. Moreover, we predicted the trends by modeling the age-period pattern in breast cancer mortality, which can be further confirmed by incorporating the cohort effects using other stochastic mortality methods. Though statistical findings in this study may differ from the actual situation, it provides a rough estimate of disease diagnosis and possible detrimental health effects. These estimates call for policymakers and governments' attention across these regions and countries to take appropriate measures to combat the disease burden.

## Conclusion

This study predicted the trends of breast cancer-related mortality in East and South Asian countries over 40 years from 1990 to 2030, showing that breast cancer-related mortality has been increasing and is expected to remain a significant public health challenge in most of these Asian countries in the next decade. Several breast cancer deaths are attributable to multiple modifiable risk factors, mainly dietary and metabolic risk factors. The increase in breast cancer-related mortality in countries with weaker public health systems and poor awareness levels may result in the reduction of life expectancy among patients with breast cancer. Therefore, improving early detection, increasing breast cancer awareness and cost-effective treatments are warranted to reduce breast cancer mortality in these Asian countries. Moreover, systematic and accurate epidemiological data collection through cancer registries is obligatory to observe the trends and assess the efficacy of respective interventions, which require collaborations between national and international organizations. Cancer awareness and community health workers education can significantly boost early detection, control, and prevention in low- and middle-income Asian countries.

## Data Availability Statement

Publicly available datasets were analyzed in this study. This data can be found at: Institute for Health Metrics and Evaluation (IHME): http://ghdx.healthdata.org/gbd-results-tool.

## Author Contributions

SM: conceptualization, data curation, formal analysis, methodology, software, validation, visualization, writing—original draft, and writing—review and editing. RS: software, validation, visualization, and writing—review and editing. SRH: software, validation, visualization, and investigation. MI and XL: data curation, validation, visualization, and investigation. N: methodology, validation, visualization, and investigation. CY: investigation, resources, validation, funding acquisition, project administration, and supervision. All authors contributed to the article and approved the submitted version.

## Funding

This study was funded by the National Key Research and Development Program of China (Grant No. 2018YFC1315302) and the National Natural Science Foundation of China (Grant Nos. 82173626 and 81773552).

## Conflict of Interest

The authors declare that the research was conducted in the absence of any commercial or financial relationships that could be construed as a potential conflict of interest.

## Publisher's Note

All claims expressed in this article are solely those of the authors and do not necessarily represent those of their affiliated organizations, or those of the publisher, the editors and the reviewers. Any product that may be evaluated in this article, or claim that may be made by its manufacturer, is not guaranteed or endorsed by the publisher.
