# Supplementary material for: Breast Cancer Mortality Trends and Predictions to 2030 and Its Attributable Risk Factors in East and South Asian Countries
Source: Front Nutr. 2022 Mar 14;9:847920. doi: 10.3389/fnut.2022.847920 (PMC8964109; doi:10.3389/fnut.2022.847920)
Supplement: Supplementary file 1 [file Data_Sheet_1.pdf]

Breast cancer mortality trends and predictions to 2030 and its attributable risk factors in East and South Asian countries

Supplementary Figure S1. Breast cancer risk factor ranking based on highest ASR of mortality in 2019 for East and South Asian regions; ASR, age-standardised rate (per 100k)

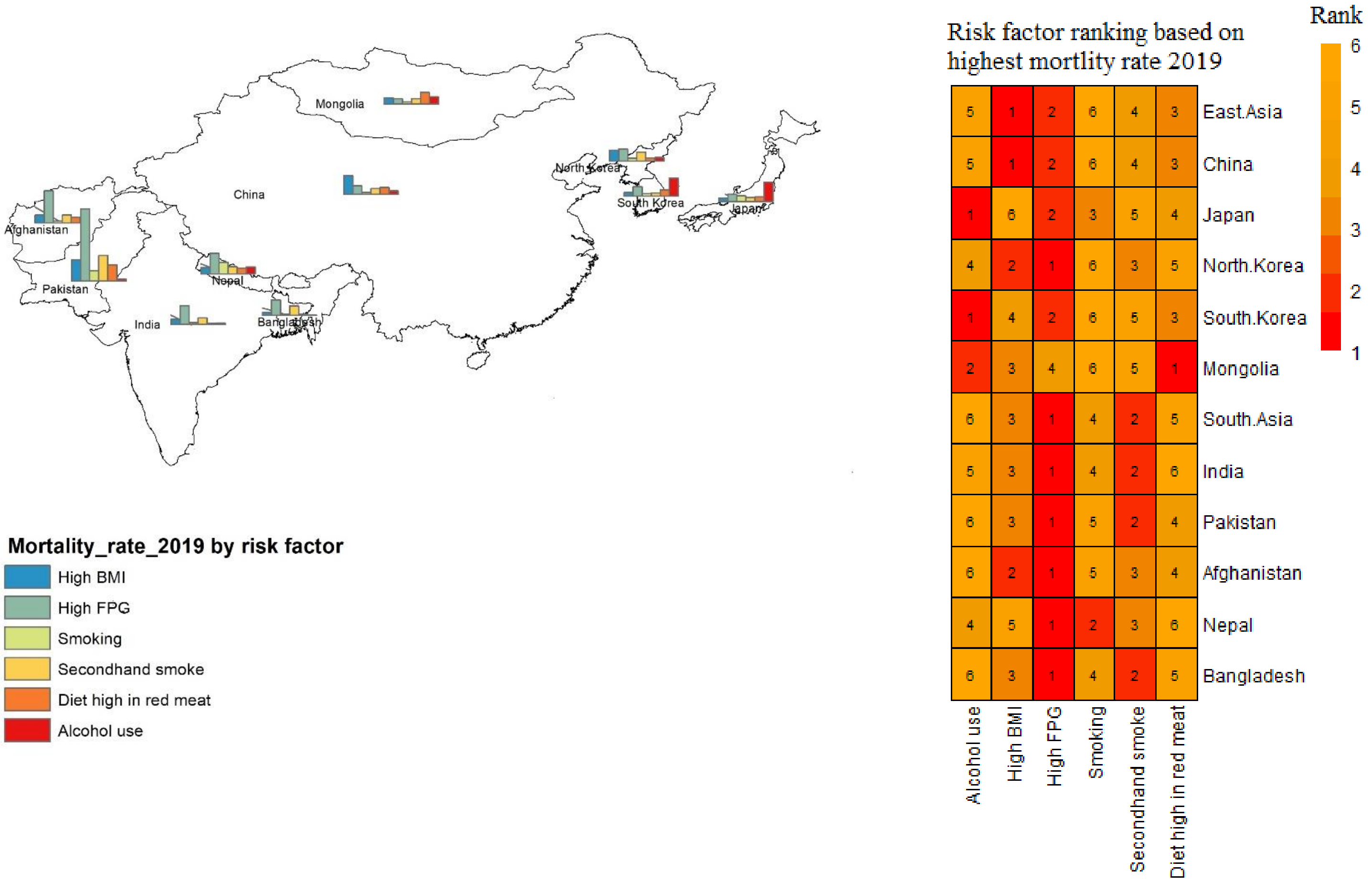

Supplementary Figure S2. Fitting of the LC model to breast cancer mortality data from East and South Asian regions

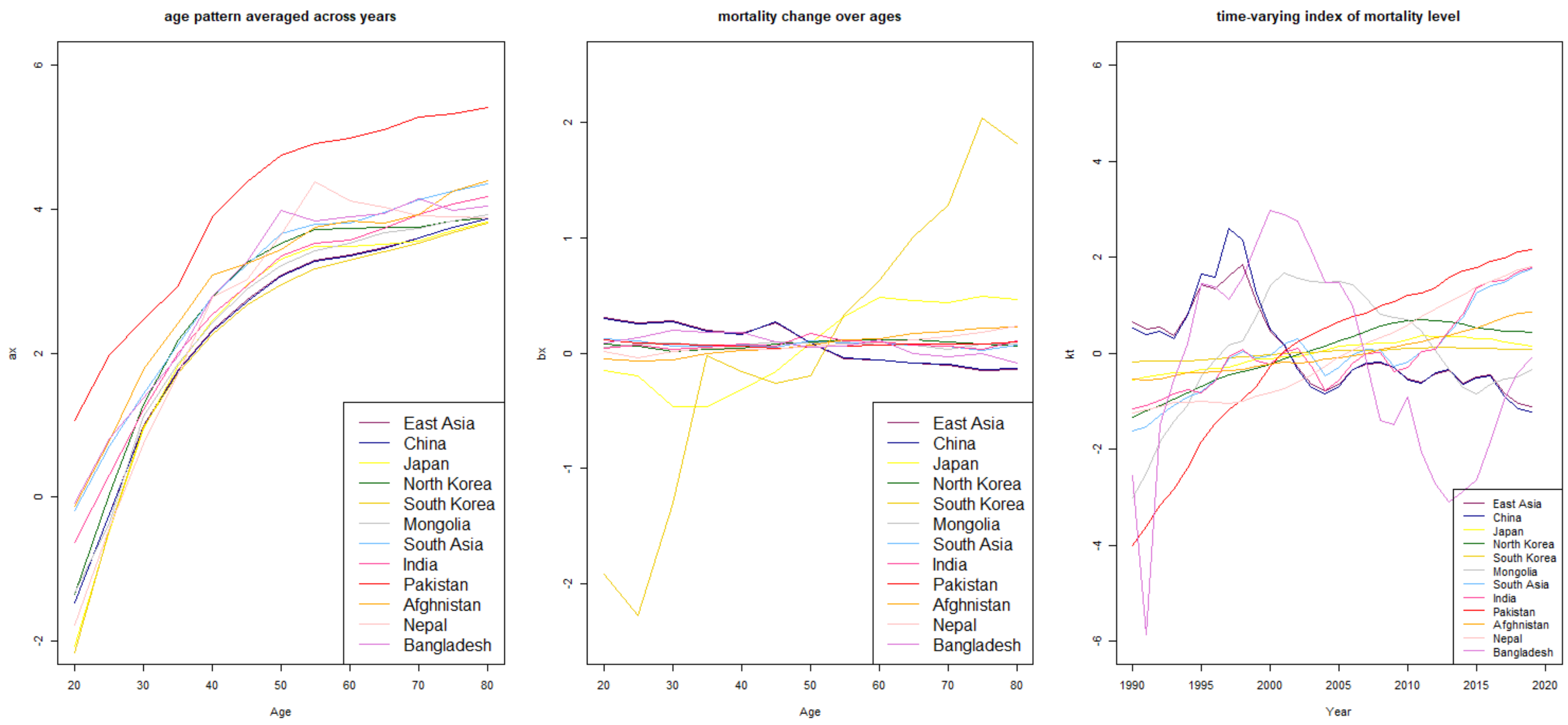

Supplementary Figure S3. Predicted trend of life expectancy from total breast cancer mortality tables, 1990-2030

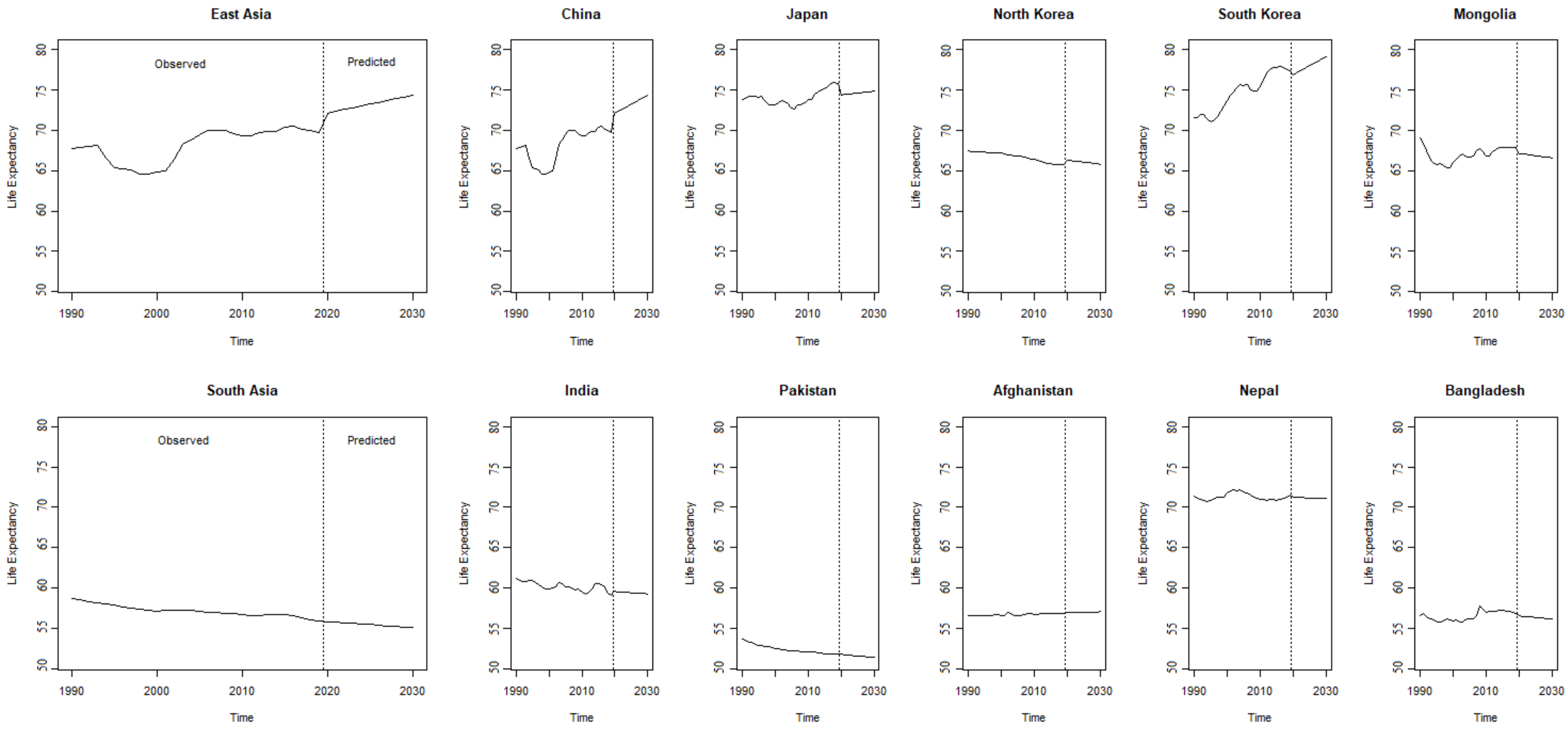

Sensitivity Analysis

Supplementary Figure S4. Fitting of the LC model and BMS model to breast cancer mortality data (1990-2019) from East and South Asian countries; **ax,** is the derived age pattern averaged across years; **bx,** stands for the sensitivity of the mortality rates to the change of kt, reflecting how fast the mortality rate changes over ages; **kt** represents the only time-varying index of mortality level

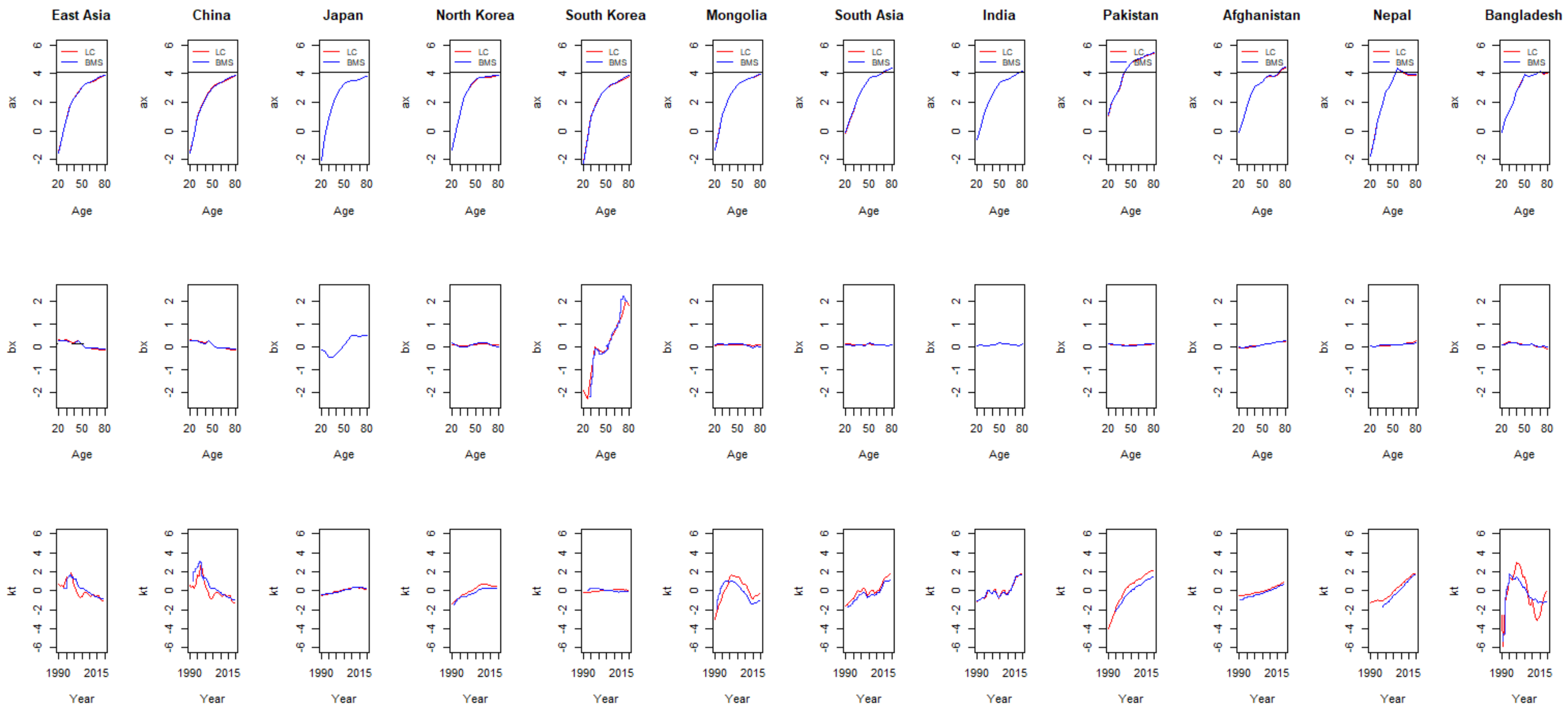

Supplementary Figure S5. Predicted death rate (log scale) from female breast cancer during 2020-2030 among East and South Asian countries; **Top panel:** rate by LC method; **Bottom panel:** rate by BMS method

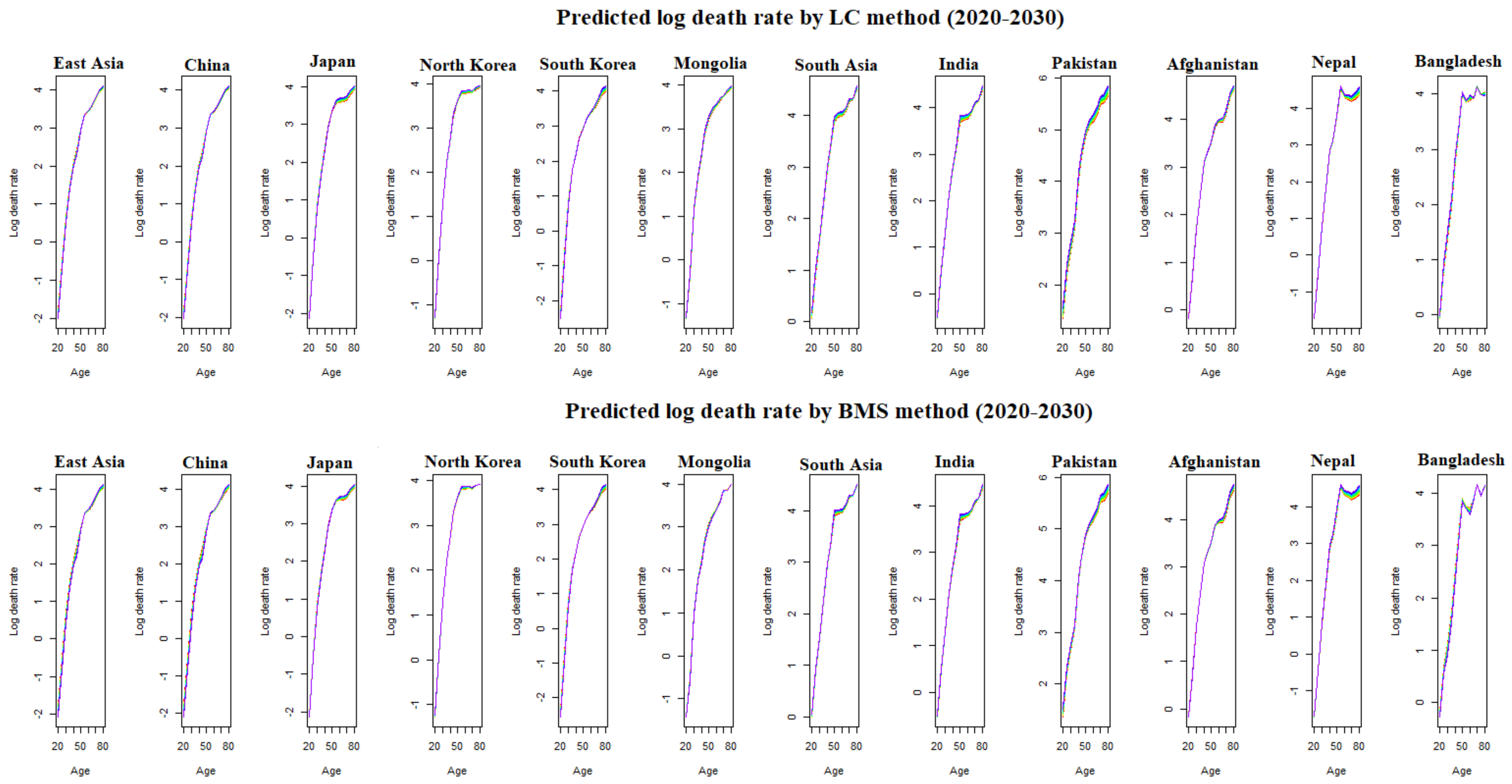

Supplementary Table S1. Estimated number of deaths, age-standardised mortality rates (ASMR) of breast cancer in 1990, 2019 and 2030 for all ages by countries

	1990		2019		2030	
**Region/Country**	**Deaths, number (95%CI)**	**ASMR, per 100k (95%CI)**	**Deaths, number (95%CI)**	**ASMR, per 100k (95%CI)**	**Deaths, number (95%CI)**	**ASMR, per 100k (95%CI)**
East Asia	43304(36019, 51019)	9.2(7.7, 10.8)	98162(79216, 120112)	9.1(7.3, 11.1)	122456(11216, 126562)	9.88(7.12, 11.4)
China	41429(34153, 49151)	9.2(7.6, 10.8)	93498(74511, 115420)	9.0(7.2, 11.1)	150867(120688, 156452)	9.59(8.41, 11.21)
Japan	7889(7544, 8093)	8.8(8.4, 9.0)	15911(13373, 17288)	10.1(9.2, 10.8)	22544(18564, 28477)	11.21(8.04, 12.62)
North Korea	1139(813, 1578)	11.0(8.0, 15.0)	2347(1726, 3125)	12.9(9.5, 17.4)	5562(4478, 6100)	14.01(12.35, 16.80)
South Korea	1419(1344, 1502)	7.6(7.2, 8.1)	4063(3582, 4548)	8.7(7.7, 9.7)	6163(5622, 7108)	9.8(7.9, 10.01)
Mongolia	50(38, 65)	8.5(6.4, 10.9)	139(101, 190)	9.9(7.3, 13.2)	202(194, 217)	10.21(8.06, 12.91)
South Asia	39454(32066, 45556)	13.4(10.6, 15.7)	125312(103075, 149357)	16.8(13.9, 20.0)	199254(180244, 230546)	18.07(9.21, 16.02)
India	25779(21135, 30096)	10.8(8.7, 12.7)	82099(63114, 104727)	13.6(10.5, 17.3)	110212(98412, 124365)	14.07(11.01, 16.41)
Pakistan	9290(6592, 12742)	33.2(23.2, 46.3)	31176(23333, 42602)	51.9(39.0, 69.7)	77874(49684, 87564)	53.89(42.30, 65.10)
Afghanistan	501(389, 642)	13.8(10.9, 17.5)	1281(964, 1673)	16.5(12.5, 21.3)	2390(1999, 2530)	18.41(11.82, 20.22)
Nepal	718(473, 1009)	13.5(8.7, 18.8)	2231(1654, 2973)	18.1(13.4, 23.8)	3754(2146, 4271)	19.99(14.50, 24.01)
Bangladesh	3650(2725, 4692)	14.7(11.1, 18.7)	9771(7661, 12279)	14.5(11.5, 18.1)	15746(10412, 17842)	15.42(12.03, 19.41)

*Age-standardised mortality rates are standardised to the WHO World Standard Population

Supplementary Table S2. Percentage variation explained by LC model and BMS model to fitting on breast cancer mortality rates

	Percentage variation explained	
**Regions/Countries**	**LC model**	**BMS model**
East Asia	83.2	82.1
China	82.8	84.0
Japan	81.7	80.5
North Korea	90.9	91.2
South Korea	94.0	92.4
Mongolia	73.9	71.6
South Asia	85.5	79.8
India	74.5	74.1
Pakistan	96.8	97.3
Afghanistan	94.5	94.7
Nepal	89.2	88.8
Bangladesh	76.1	75.3

Supplementary Table S3. Error measures from LC model and BMS model to fitting on breast cancer mortality rates; **ME**, mean error; **MSE**, mean square error; **MPE**, mean percent error; **MAPE**, mean absolute percent error

**Country**			**Error Measures**			
		**Method**	**ME**	**MSE**	**MPE**	**MAPE**
East Asia	Averages across ages	LC	0.00	1.90	0.006	0.07
		CBD	0.00	1.93	0.008	0.09
	Averages across years	LC	0.27	114.12	0.44	4.17
		CBD	-0.51	126.11	0.76	4.11
China	Averages across ages	LC	0.00	3.38	0.012	0.08
		CBD	0.00	3.49	0.013	0.07
	Averages across years	LC	0.89	194.80	0.74	4.97
		CBD	-0.97	199.34	0.88	3.23
Japan	Averages across ages	LC	0.00	1.38	0.00	0.04
		CBD	-0.01	1.69	0.00	0.03
	Averages across years	LC	-0.10	78.56	0.09	2.72
		CBD	-0.87	86.2	0.07	2.08
North Korea	Averages across ages	LC	0.00	0.30	0.00	0.01
		CBD	0.00	0.31	0.00	0.01

	Averages across years	LC	0.03	17.14	0.013	0.72
		CBD	-0.01	16.09	0.00	0.61
South Korea	Averages across ages	LC	0.00	0.22	0.00	0.02
		CBD	0.00	0.14	0.00	0.02
	Averages across years	LC	-0.03	10.16	0.05	1.68
		CBD	-0.02	11.26	0.03	1.44
Mongolia	Averages across ages	LC	0.00	5.49	0.00	0.07
		CBD	-0.05	5.31	0.001	0.03
	Averages across years	LC	0.7	284.7	0.45	4.48
		CBD	-2.72	262	0.108	2.14
South Asia	Averages across ages	LC	0.00	1.45	0.00	0.02
		CBD	-0.00	1.83	0.00	0.02
	Averages across years	LC	0.11	68.24	0.05	1.26
		CBD	-0.02	77.40	0.03	1.13
India	Averages across ages	LC	0.00	1.62	0.00	0.03
		CBD	0.00	1.63	0.00	0.03
	Averages across years	LC	0.12	76.77	0.08	1.75
		CBD	0.13	76.4	0.08	1.75
Pakistan	Averages across ages	LC	0.00	9.04	0.00	0.02
		CBD	0.00	10.02	0.00	0.01
	Averages across years	LC	0.12	170.11	0.03	1.19
		CBD	-0.16	188.26	0.04	1.64
Afghanistan	Averages across ages	LC	0.00	0.05	0.00	0.00
		CBD	0.00	0.06	0.00	0.00
	Averages across years	LC	0.00	6.64	0.00	0.57
		CBD	-0.02	8.23	0.00	0.66
Nepal	Averages across ages	LC	0.00	1.58	0.00	0.03
		CBD	0.00	1.84	0.00	0.01
	Averages across years	LC	-0.13	82.08	0.06	1.79
		CBD	-0.12	86.48	0.09	1.72
Bangladesh	Averages across ages	LC	0.00	38.80	0.00	0.12
		CBD	-0.04	53.62	0.00	0.14
	Averages across years	LC	-4.87	13.23	0.04	8.59
		CBD	-3.97	16.87	0.06	7.61
